# Comparison of Sum Absolute QRST Integral, and Temporal Variability in Depolarization and Repolarization, Measured by Dynamic Vectorcardiography Approach, in Healthy Men and Women

**DOI:** 10.1371/journal.pone.0057175

**Published:** 2013-02-22

**Authors:** Sanjoli Sur, Lichy Han, Larisa G. Tereshchenko

**Affiliations:** 1 Whiting School of Engineering, The Johns Hopkins University, Baltimore, Maryland, United States of America; 2 The Division of Cardiology, Department of Medicine, Johns Hopkins Hospital, Baltimore, Maryland, United States of America; University of Adelaide, Australia

## Abstract

**Background:**

Recently we showed the predictive value of sum absolute QRST integral (SAI QRST) and repolarization lability for risk stratification of sudden cardiac death (SCD) in heart failure patients. The goal of this study was to compare SAI QRST and metrics of depolarization and repolarization variability in healthy men and women.

**Methods:**

Orthogonal ECGs were recorded at rest for 10 minutes in 160 healthy men and women (mean age 39.6±14.6, 80 men). Mean spatial TT′ angle, and normalized variances of T loop area, of spatial T vector amplitude, of QT interval and Tpeak-Tend area were measured for assessment of repolarization lability. Normalized variances of spatial QRS vector and QRS loop area characterized variability of depolarization. In addition, variability indices (VI) were calculated to adjust for normalized heart rate variance. SAI QRST was measured as the averaged arithmetic sum of areas under the QRST curve.

**Results:**

Men were characterized by shorter QTc (430.3±21.7 vs. 444.7±22.2 ms; P<0.0001) and larger SAI QRST (282.1±66.7 vs.204.9±58.5 mV*ms; P<0.0001). Repolarization lability negatively correlated with spatial T vector amplitude. Adjusted by normalized heart rate variance, QT variability index was significantly higher in women than in men (−1.54±0.38 vs. −1.70±0.33; P = 0.017). However, in multivariate logistic regression after adjustment for body surface area, QTc, and spatial T vector amplitude, healthy men had 1.5–3 fold higher probability of having larger repolarization lability, as compared to healthy women (T vector amplitude variability index odds ratio 3.88(95%CI 1.4–11.1; P = 0.012).

**Conclusions:**

Healthy men more likely than women have larger repolarization lability.

## Introduction

Sudden cardiac death (SCD) is the most frequent mode of cardiovascular death among both men and women [1]. Further improvement of SCD risk stratification is needed. Recently we developed VCG approach to the assessment of temporal repolarization lability [three-dimensional ECG (3D ECG [2]–[4])], VCG interloop distance (ID) metric [5] and ECG parameter sum absolute QRST integral (SAI QRST [6]–[8]), and showed their predictive value for life-threatening ventricular arrhythmia in patients with systolic heart failure [2], [6]–[9]. However, our risk markers have not been measured in healthy individuals. The goal of this study was to compare repolarization lability as measured by dynamic VCG approach, interloop distance, and SAI QRST in healthy males and females across different ages, along with other well-established ECG risk markers, such as spatial ventricular gradient (SVG), spatial QRS-T angle, and QRS and QT intervals.

## Methods

### Study population

All study participants signed consent forms before entering the study. The research involved analysis of existing data, specifically digital electrocardiograms of the Intercity Digital Electrocardiogram Alliance (IDEAL) study [10], database of high resolution orthogonal ECGs, provided by NIH-funded The Telemetric and Holter ECG Warehouse (THEW) initiative [11] under the data usage agreement. Data have been recorded in such a manner that the subjects cannot be identified, directly or through identifiers linked to the subject. Therefore, the proposed research meets the criteria for Exemption number 4, or else is exempt under 45 CFR 46.101 (b)(4) from all 45 CFR part 46 requirements. All authors reside in the U.S., no one conducted this research outside of the country of residence. The de-identified dataset that accompanied digital ECG recording contained information on age, gender, race, height, weight, systolic and diastolic blood pressure, and smoking status. Study population was previously described [10], [12]. Healthy status of participants was confirmed by the absence of a history of any chronic illnesses, normal physical examination, and normal 12 leads ECG in sinus rhythm. Echocardiogram and ECG exercise testing was performed to confirm healthy status of participants if clinically indicated. Ten minutes of high resolution modified (5^th^ intercostal space) Frank orthogonal XYZ ECG recordings were acquired using the SpaceLab-Burdick digital Holter recorder (SpaceLab-Burdick, Inc., Deerfield, WI) with 1000 Hz sampling frequency and 4.88 µV amplitude resolution. Only adult subjects 18 years and older were included in our analysis. Body surface area (BSA) was calculated using the Mosteller formula.

### Orthogonal ECG analysis

Customized MATLAB (MathWorks, Inc, Natick, MA) software for automated ECG analysis was developed in Tereshchenko's laboratory. Noise and premature atrial and ventricular beats, and one subsequent sinus beat were excluded from analysis. Only sinus beats were analyzed. Software automatically detected and marked fiducial points (onset of Q or R wave, peak of R, J-point, peak of T wave and end of T wave) on each lead (X,Y,Z). Then ECGs were reviewed by 3 investigators (SS, LH, LGT) to ensure appropriate fiducial point detection. ECGs were automatically re-analyzed if fiducial points were placed incorrectly. Improved detection of fiducial points was achieved by adjusting detection algorithms (adjusting window of interest or adjusting particular threshold). Method of Zong et al was used to measure QRS width [13] and QT interval duration [14]. QT interval averaged over 3 minutes was corrected for heart rate (HR) using the Bazett formula [15].

### Sum absolute QRST integral (SAI QRST) and spatial ventricular gradient (SVG)

Sum absolute QRST integral (SAI QRST) was measured as the arithmetic sum of areas under the QRST curve, averaged during a 3-minute epoch as previously described [6], [7]. SAI QRST was calculated according to equation:
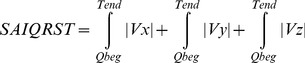
(1)


Similarly, sum absolute QRS integral was measured as the arithmetic sum of areas under the QRS curve on 3 orthogonal leads:
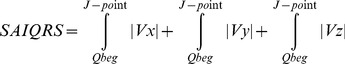
(2)


Sum absolute JT integral was measured as the arithmetic sum of areas under the JT curve on 3 orthogonal leads
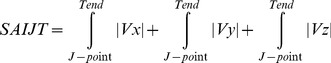
(3)


Spatial ventricular gradient (SVG) was measured as described by Burch et al and Cortez et al [16], [17]. The orthogonal components of SVG were calculated by integrating each lead over the QT interval. Posterior (Z), downward (Y), and leftward (X) directions were designated as positive. The left end of the X axis and the anterior end of the Z axis were, respectively, designated as 0° and +90° for the azimuth. The inferior end of the Y axis was designated as 0° for the elevation. SVG was calculated according to equation:
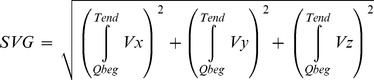
(4)


Azimuth and elevation of SVG were calculated as following:
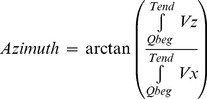
(5)

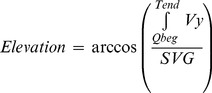
(6)


### 3-dimensional electrocardiography, or dynamic vectorcardiography

**Figure 1:**
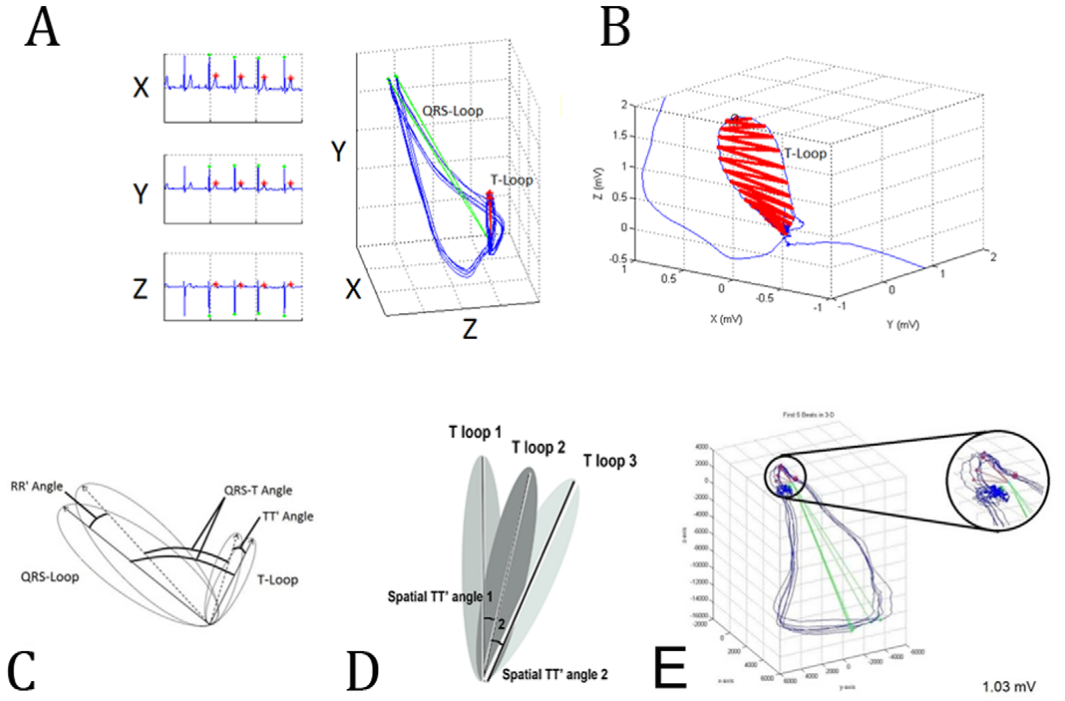
Dynamic VCG (3-dimensional ECG) method. (A) Representative example of recorded orthogonal ECG (XYZ leads), and (B) constructed QRS and T loops with detected fiducial points (R peak, T peak, point of origin, spatial T vector, spatial QRS vector); (C) Magnified image of T loop illustrates T loop area calculations; (D) Scheme of the spatial TT′ angle measurement as the angle between two consecutive T vectors. Similarly, the spatial RR′ angle is the angle between two consecutive QRS vectors; (E) Representative example demonstrating interloop distance measurement; (F) magnified interloop distance view.

Epochs of 3 minutes were selected and corrected for baseline wandering. Fiducial points (peak of spatial QRS vector, peak of spatial T vector, and the origin point) were detected automatically. VCG loops with automatically detected marked fiducial points were reviewed by 3 investigators (SS, LH, LGT) to ensure accuracy. The origin point was detected as halfway between the two points that were closest in space but significantly separated in time. The peak of the spatial QRS vector and the peak of the spatial T vector were detected as the furthest points from the origin point in the QRS-loop and T-loop respectively (Figure 1). Mean amplitudes of spatial T vector and spatial QRS vectors were calculated.

The spatial TT′ angle was calculated as the angle between two consecutive T vectors using the definition of the inner product. Mean spatial TT′ angle was calculated as a measure of beat-to-beat spatial T-axis variability. Mean spatial RR′ angle was calculated between 2 consecutive spatial QRS vectors (Figure 1C). The areas of the T-loop and of the QRS-loop were calculated on the dynamic main plane using successive triangles along the loop from the peak to the origin point (Figure 1B). There was no single pre-defined plane to measure loop area. Area of each triangle was measured on the plane defined by each respective triangle. Each triangle was defined by 2 consecutive points on one side of the loop, and one point on the opposite side of the loop. The side with 2 points alternated sides. Distance between consecutive points on one side of the loop was 1 ms. Variability of spatial T vector magnitude, spatial QRS vector magnitude, T-loop area and QRS-loop area were measured as their respective variances. Additionally, convex hull volumes of the spatial QRS vector peaks cloud and T vector peaks cloud were measured, and the ratio of the T peaks cloud volume to the R peaks cloud volume was calculated as previously described [2], [3].

Interloop distance (Figure 1E) was measured on 3D ECG for each cardiac cycle between two points on the QRS and T loops (point P1 and point P2). In the window between the peak of R and the onset of P wave of consecutive beat, the algorithm searched for 2 points separated as much as possible in time, and then minimized the difference in 3D space. Interloop distance was calculated according to equation:
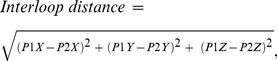
(7)where P1X is Point 1 on X lead, P2X is Point 2 on X lead, P1Y is point 1 on Y lead, P2Y is Point 2 on Y lead, P1Z is Point 1 on Z lead, P2Z is Point 2 on Z lead.

### Temporal variability of repolarization

The spatial TT′ angle was calculated as the average angle between two consecutive T vectors using the definition of the inner product. For example, 1^st^ TT′ angle was measured between 2^nd^ and 3^rd^ beats T vectors, 2^nd^ TT′ angle was measured between 3^rd^ and 4^th^ beats T vectors, 3^rd^ TT′ angle was measured between the 4^th^ and 5^th^ T vectors, etc. Then averaged spatial TT′ angle was calculated.

Measured variances of spatial T vector magnitude and of T-loop area were normalized by respective mean values and log-transformed. Normalized T-loop area variance (TareaVN) was calculated according to the equation:
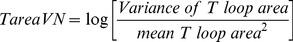
(8)


Normalized T amplitude variance (TampVN) was calculated according to the equation:
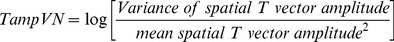
(9)


Repolarization lability was also measured as the root mean square successive difference (rMSSD) of the area under the curve from T-peak to T-end series (TpTe Area rMSSD). Normalized Tpeak-Tend area variance (TpTeAVN) was calculated according to equation:
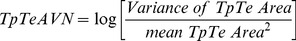
(10)


In addition, QT variance was calculated as the variance of the QT interval. QT variance was first calculated on each lead and then averaged across the 3 leads. Normalized QT variance (QTVN) was calculated according to equation:
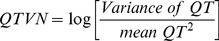
(11)


### Spatial QRS-T angle and its variability

Spatial “peak” QRS-T angle was measured as an angle between spatial QRS vector and spatial T vector on each beat and averaged over 3 minutes (Figure 1). Variance of spatial peak QRS-T angle was calculated. Normalized variance of spatial peak QRS-T angle (QRS-TVN) was calculated according to equation:
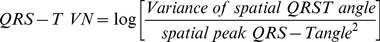
(12)


### Temporal variability of depolarization

Spatial QRS vector magnitude and area of QRS-loop were measured on each beat. Mean and variance of spatial QRS vector magnitude and QRS-loop area were calculated. Normalized QRS-loop area variance (QRSareaVN) was calculated according to the equation:
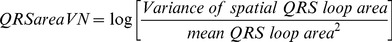
(13)


Normalized QRS amplitude variance (QRSampVN) was calculated according to the equation:
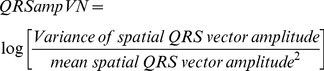
(14)


### Depolarization and repolarization variability indices

In order to quantify heart rate variability, heart rate variance was measured and normalized by mean heart rate, according to the equation:
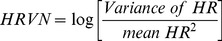
(15)


In order to uniformly adjust temporal variability parameters by heart rate variability, normalized variability metrics were adjusted by normalized heart rate variance, following an example of QT variability index (QTVI) calculation as proposed by Berger et al [18], according to the following equations for spatial QRS vector variability index (16), QRS loop area variability index (17), spatial T vector variability index (18), T loop variability index (19), and spatial QRS-T angle variability index (20).
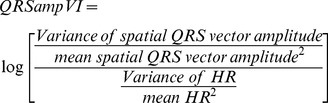
(16)

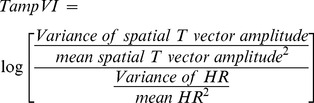
(17)

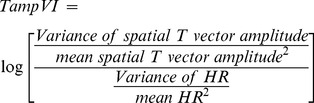
(18)

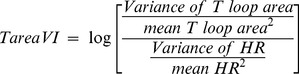
(19)

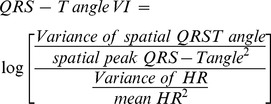
(20)


### Statistical analysis

Statistical analysis was conducted using Stata 12 (StataCorp, College Station, TX). All of the numerical parameters are presented in the form mean ± standard deviation for normally distributed variables, and as median and interquartile range (IQR) for variables with skewed distribution. The normally distributed numerical variables in men and women were compared using a standard t-test, while Pearson's chi-squared test was used to compare categorical variables. The Wilcoxon rank-sum test was used to compare variables which demonstrated a skewed distribution. Pairwise correlations have been studied. Spearman's rank correlation coefficient rho was calculated if non-linear relationships were noticed. Multiple logistic regression analyses were performed to determine specific ECG predictors of gender after adjustment for age, race and body size (height, weight, BSA, BMI). Gender served as an outcome in this analysis. P-values less than 0.05 were considered significant.

## Results

### Study population characteristics

We analyzed data of 181 IDEAL individuals. After exclusion of individuals younger than 18 years and those with noisy ECGs, data of remaining 160 study participants (80 men and 80 women) were further analyzed. Comparison of the demographics of the two gender populations is summed up in Table 1. As expected, women were significantly shorter and lighter and had smaller BSA. However, no significant difference in BMI was observed. Systolic and diastolic blood pressure was lower in women (Table 1).

**Table 1:** Comparison of demographics in healthy men and women.

	Male (N = 80)	Female (N = 80)	P
Age±SD, y	38.8±13.0	40.4±16.2	0.478
Whites, n(%)	75(94)	74(93)	0.349
**Height±SD, cm**	177.1±7.9	162.8±6.0	**<0.0001**
**Weight±SD, kg**	78.2±11.5	62.5±14.2	**<0.0001**
**Systolic BP±SD, mmHg**	119.3±10.8	113. 8±12.5	**0.0058**
**Diastolic BP±SD, mmHg**	76.9±7.7	73.4±8.3	**0.01**
BMI±SD, kg/m^2^	24.9±2.8	23.7±5.7	0.097
**BSA±SD**,	1.96±0.17	1.67±0.18	**<0.0001**
Smoking, n(%)	27(33.8)	22(27.5)	0.325

BP = blood pressure; SD = standard deviation; BMI = body mass index; BSA = body surface area.

### Comparison of ECG and VCG parameters in healthy men and women

**Figure 2:**
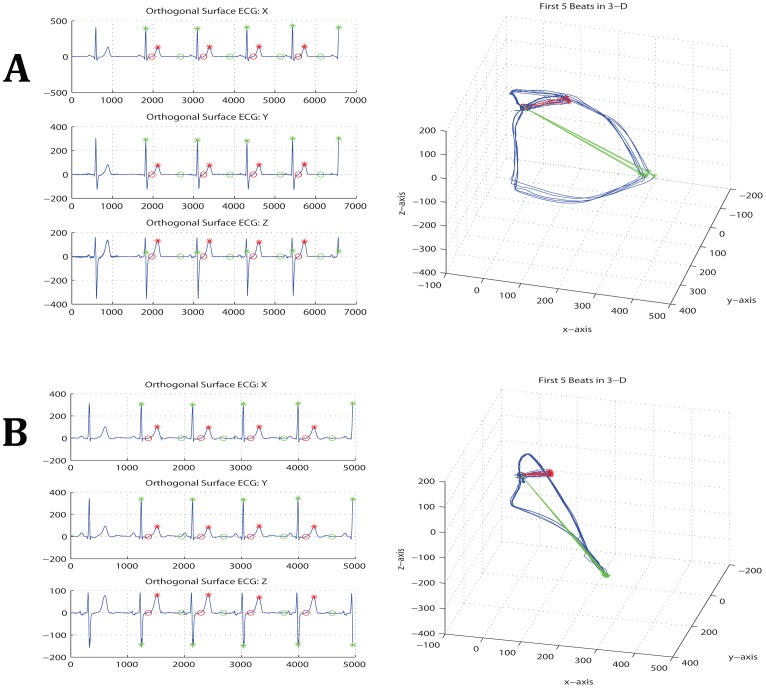
Representative examples of 3-dimensional ECG. 3 minutes epoch ECG show spatial T-to-T′ angles in a Male participant (**A**) as compared with a Female participant (**B**) of the same age (42 y).

Representative examples of ECG in XYZ leads and VCG loops for a man and a woman of similar age (42 y.) are presented in Figure 2 and Movie S1 and S2. Table 2 provides a comparison of ECG and VCG parameters in both gender populations. As one would expect, males had slower HR, wider QRS, larger magnitudes of spatial QRS vector and spatial T vector, larger areas of QRS and T loops, larger spatial QRS-T angle, and larger SAI QRST, SAI QRS and SAI JT (Table 2). SVG, its elevation and azimuth were significantly larger in men as well. As anticipated, QTc was significantly longer in healthy adult women, as compared to the healthy adult men. SVG strongly correlated with SAI QRST in men (r = 0.77; P<0.0001) and women (r = 0.85; P<0.0001).

**Table 2:** Comparison of measured ECG and dynamic VCG Parameters in healthy men and women.

ECG parameter	Males (N = 80)		Females (N = 80)		P
	Mean±SD	95%CI	Mean±SD	95%CI	
**Mean heart rate±SD,bpm**	62.8±9.6	60.7–65.0	67.6±11.2	65.1–70.1	**0.005**
**Heart rate variance median (IQR), ms^2^**	3.35(1.54–8.96)	0.86–30.08	5.50(2.92–9.64)	0.71–42.81	**0.018**
HRVN±SD	−6.90±1.20	−7.17 to −6.63	−6.68±1.13	−6.93 to −6.43	0.234
**QRS width±SD, ms**	98.0±7.5	96.0–99.9	92.9±6.5	91.3–94.5	**0.0001**
**QTc±SD, ms**	396.3±20.8	390.9–401.6	409.9±24.3	403.9–415.9	**0.0009**
SAI QRST±SD,mV*ms	274.6±66.6	257.4–291.8	200.2±55.0	186.6–213.8	**<0.0001**
SVGmagnitude±SD,mV*ms	103.6±31.8	96.6–110.7	83.5±29.2	77.0–90.0	**0.0001**
**SVG azimuth±SD, deg**	11.7±26.8	5.6–14.6	−1.1±34.0	−8.5–6.4	**0.0091**
**SVG elevation±SD, deg**	58.1±25.0	52.5–63.7	49.1±26.00	43.3–54.9	**0.027**
Interloop distance median (IQR), mV	0.019(0.013–0.28)	0.019–0.026	0.014(0.010–0.026)	0.017–0.023	0.107
**Spatial peak QRS-T angle±SD, deg**	56.8±33.9	49.2–64.3	47.0±26.1	41.1–52.8	**0.042**
**Mean spatial QRS vector amplitude±SD, mV**	2.53±0.67	2.38–2.68	2.18±0.62	2.04–2.31	**0.0007**
SAI QRS±SD,mV*ms	139.6±34.4	130.7–148.5	104.1±25.7	97.8–110.5	**<0.0001**
QRSampVN±SD	−7.64±1.00	−5.7–(−5.0)	−7.38±1.09	−7.6–(−7.1)	0.112
QRSareaVN±SD	−6.12±0.89	−6.3–(−5.9)	−5.92±0.93	−6.4–(−4.2)	0.166
Mean RR′angle±SD, deg	2.5±1.1	2.1–3.6	2.4±1.1	2.1–2.6	0.607
**Mean QRS loop area±SD,mV^2^**	633.5±335.6	559.1–707.5	404.8±215.2	356.9–452.7	**<0.0001**
QRS-TVN±SD	−5.35±1.57	−5.7–(−5.0)	−4.93±1.23	−5.2–(−4.7)	0.063
SAI JT±SD,mV*ms	135.0±42.8	123.9–146.0	96.0±36.7	86.9–105.1	**<0.0001**
**QTVN±SD**	0.225(0.130–0.384)	−8.5–(−8.1)	0.408(0.232–0.815)	−7.9–(−7.5)	**0.0001**
TampVN±SD	−6.22±0.83	−6.4–(−6.0)	−5.99±0.94	−6.2–(−5.8)	0.103
**Mean TT′±SD, deg**	2.9±1.2	2.7–3.4	3.7±1.7	3.4–4.3	**0.0008**
**Mean spatial T-vector amplitude** ±SD, mV	0.80±0.25	0.74–0.86	0.60±0.24	0.54–0.65	**<0.0001**
**Mean T loop area±SD,mV^2^**	172.6±102.8	149.7–195.2	99.4±78.5	81.9–116.9	**<0.0001**
**TareaVN±SD**	−4.73±0.91	−4.9–(−4.5)	−4.39±0.99	−4.6–(−4.2)	**0.026**
**TpTeAVN±SD**	0.006±0.004	0.006–0.007	0.010±0.0099	0.008–0.012	**0.0021**
T/R peaks cloud ratio, median(IQR)	0.19(0.11–0.34)	0.21–0.33	0.20(0.10–0.35)	0.01–0.37	0.757

**Figure 3:**
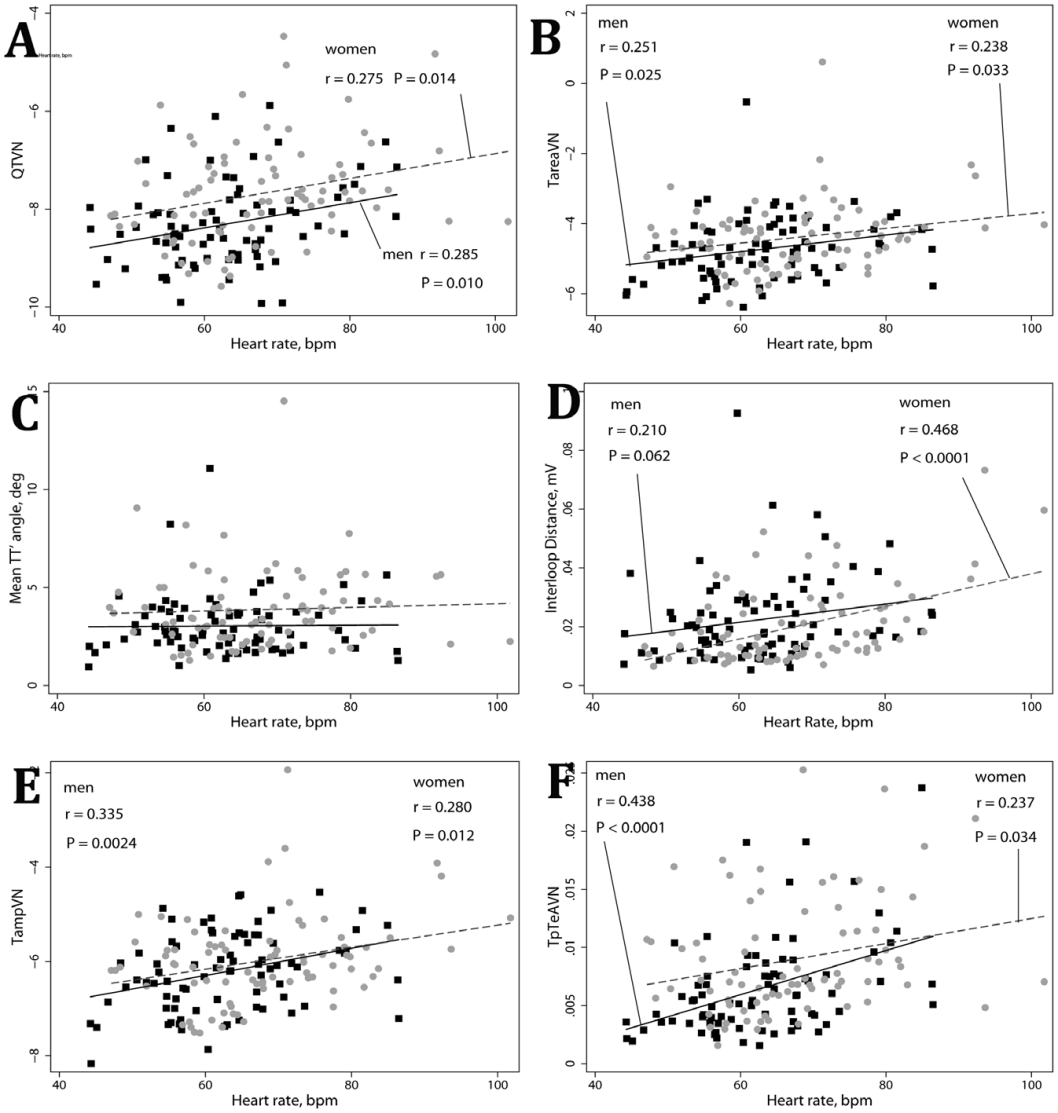
Correlations between ECG metrics and heart rate. Scatterplots of repolarization lability parameters (A) QTVN, (B) Normalized variance of T loop area TareaVN, (C) spatial TT′ angle, (D) interloop distance, (E) normalized variance of spatial T vector amplitude TampVN, (F) normalized variance of Tpeak-Tend area TpTeAVN (Y) against heart rate (X). Black squares represent male data points. Red circles represent female data points. A line of best fit is shown separately for men (black solid line) and women (red dashed line). Quadratic fit is used if non-linear relationships.

There was a trend towards larger interloop distance in men (Table 2). Interestingly, in men interloop distance did not correlate with HR, however significant positive correlation between HR and interloop distance was found in women (Figure 3D).

### Comparison of temporal variability of depolarization and repolarization in healthy men and women

As variability of ECG or VCG parameters depends on both the value of the studied parameter, and on heart rate and heart rate variability, we systematically applied both approaches: first we normalized variance of studied metric, and then we adjusted it by normalized heart rate variance. Heart rate variance was higher in women (Table 2), whereas no difference in normalized heart rate variance between genders was observed. Temporal variability of depolarization did not differ in men and women, as measured by normalized QRS loop area variance, normalized variance of spatial QRS vector amplitude (Table 2), as well as by variability indices of QRS loop area and spatial QRS vector amplitude (Table 3).

**Table 3:** Comparison of variability indices in healthy men and women.

Parameter	Male (N = 80)	Female (N = 80)	P
QRS vector amplitude variability index (SD)	−0.32(0.56)	−0.30(0.57)	0.830
QRS loop area variability index (SD)	0.34(0.55)	0.33(0.51)	0.920
QRS-T angle variability index (SD)	0.67(0.72)	0.76(0.73)	0.46
QT interval variability index (SD)	−1.70(0.33)	−1.54(0.38)	0.017
T vector amplitude variability index (SD)	0.30(0.59)	0.30(0.54)	0.970
T loop area variability index (SD)	0.94(0.56)	0.99(0.55)	0.560

**Figure 4:**
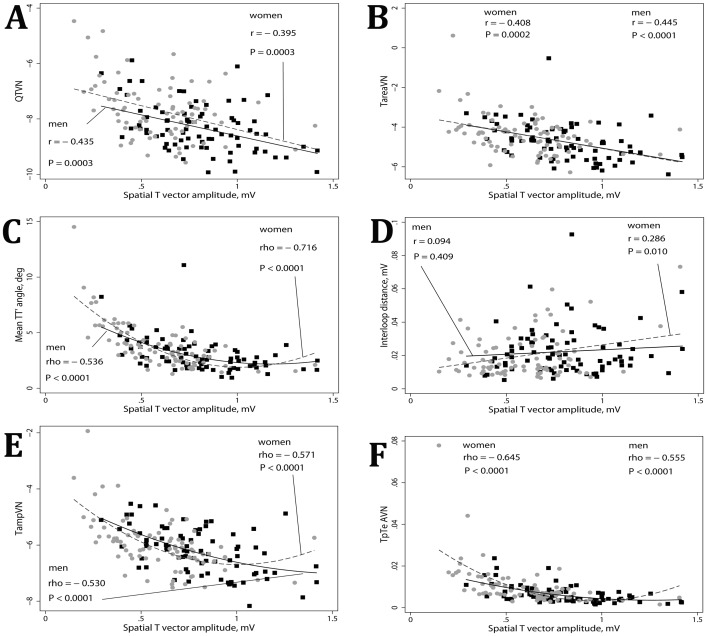
Correlations between ECG metrics and spatial T vector amplitude. Scatterplots of repolarization lability parameters (A) QTVN, (B) Normalized variance of T loop area TareaVN, (C) spatial TT′ angle, (D) interloop distance, (E) normalized variance of spatial T vector amplitude TampVN, (F) normalized variance of Tpeak-Tend area TpTeAVN (Y) against spatial T vector amplitude (X). Black squares represent male data points. Red circles represent female data points. A line of best fit is shown separately for men (black solid line) and women (red dashed line). Quadratic fit is used if non-linear relationships.

In univariate analysis (Table 2) repolarization lability (normalized beat-to-beat variability of spatial T axis angle, T-loop area, Tpeak-Tend area, QT interval, and spatial TT′ angle) was significantly larger in healthy women, as compared to healthy men. Repolarization lability negatively correlated with spatial T vector amplitude: the smaller T vector, the larger repolarization lability (Figure 4). All but one repolarization lability metrics correlated with HR (Figure 3A–B and 3E–F). Mean TT′ angle did not correlate with HR (Figure 3C). After adjustment for normalized heart rate variance, only QTVI was significantly higher in women (Table 3), whereas adjusted by normalized heart rate variance variability indices of spatial T vector amplitude and T loop area did not differ in men and women (Table 3).

In order to discover which specific ECG and/or VCG parameters determine gender differences after adjustment for age, race, body constitution (BSA or BMI), and systolic blood pressure, we ran multiple logistic models with sex as an outcome, and we included ECG and VCG parameters as predictors (Table 4).Final models with optimal fit are presented in Table 4. After adjustment for BMI, systolic blood pressure, and QTc, both SVG and SAI QRST determined gender. Interestingly, larger SAI QRST, but smaller SVG was associated with male sex. After adjustment for BSA, heart rate, QTc, and amplitude of spatial T vector (or QRS vector, accordingly), adjusted by heart rate variability metrics of temporal repolarization variability (spatial T vector amplitude variability index, T loop variability index, spatial TT′ angle), and depolarization variability (spatial QRS vector amplitude variability index) were associated with sex. Surprisingly, larger repolarization lability was associated with male sex (Table 4).

**Table 4:** Multiple logistic regression models predicting male sex.

	Predictor	Odds Ratio (95% CI)	P value
Model 1	Systolic blood pressure, mmHg	1.07(1.02–1.12)	0.007
	Body mass index, kg/m^2^	1.13(1.00–2.27)	0.042
	SVG, mV*ms	0.96(0.93–0.99)	0.008
	SAI QRST, mV*ms	1.04(1.02–1.06)	<0.0001
	QTc, ms	0.97(0.95–0.99)	0.014
Model 2	Body surface area, m^2^	35967(1306–990474)	<0.0001
	QTc, ms	0.956(0.932–0.980)	<0.0001
	Spatial T vector amplitude, mV	382.5(13.9–1053.4)	<0.0001
	Spatial TT′ angle, deg	1.52(1.01–2.29)	0.046
	Normalized heart rate variance	0.65(0.41–1.03)	0.069
Models 3	Spatial TT′ angle, deg	1.52(1.01–2.29)	0.046
	Spatial T vector amplitude VI (eq. 18)	3.88(1.35–11.14)	0.012
	T loop area VI (eq. 19)	3.04(1.10–8.46)	0.033
	QTVI	1.45(0.24–8.76)	0.683
Models 4	Spatial QRS vector amplitude VI (eq. 16)	2.69(1.02–7.07)	0.045
	QRS loop area VI (eq. 17)	2.31(0.85–6.24)	0.099
	QRS-T angle VI (eq. 20)	1.42(0.72–2.79)	0.313

Models #3 for variability indices (calculated per equations 18,19) and QTVI were adjusted by QTc, body surface area, and spatial T vector amplitude. Model 3 for spatial TT′ angle in addition was adjusted by normalized heart rate variance. Models #4 for variability indices (calculated per equations 16, 17, and 20) were adjusted by QTc, body surface area, and spatial QRS vector amplitude.

**Figure 5:**
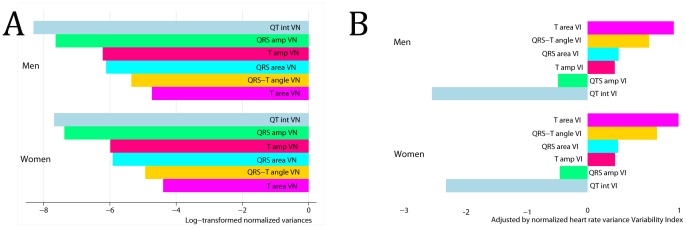
Comparison of the degree of depolarization and repolarization variability. Bar graphs of the mean values of (A) log-transformed normalized variances and (B) adjusted by normalized heart rate variance depolarization and repolarization variability indices in Men and Women. QT int VN = normalized variance of QT interval. QRSampVN = normalized variance of spatial QRS vector amplitude. QRSareaVN = normalized variance of QRS loop area. QRS-T angle VN = normalized variance of spatial QRS-T angle. TareaVN = normalized variance of T loop area. TampVN = normalized variance of spatial T vector amplitude. QT int VI = QT variability index. QRS amp VI = spatial QRS vector amplitude variability index. QRS area VI = QRS loop area variability index. TareaVI = T loop area variability index. T amp VI = spatial T vector amplitude variability index. QRS-T angle VI = spatial QRS-T angle variability index.

In addition, we compared degree of normalized and adjusted variability across the spectrum of studied uniformly calculated parameters (Figure 5A and B). In Figure 5 calculated indices are presented in the increasing order. Numerically area of T loop demonstrated the largest normalized variability, whereas QT interval variability was the smallest. The same pattern became even more obvious after adjustment for normalized HR variance in variability indices (Figure 5B). Numerically normalized variances of spatial T vector amplitude, spatial QRS-T angle, QRS and T areas were larger, than normalized HR variance. However, normalized variances of spatial QRS vector amplitude and QT interval were smaller, than normalized HR variance.

### Effect of age on ECG and VCG parameters

**Figure 6:**
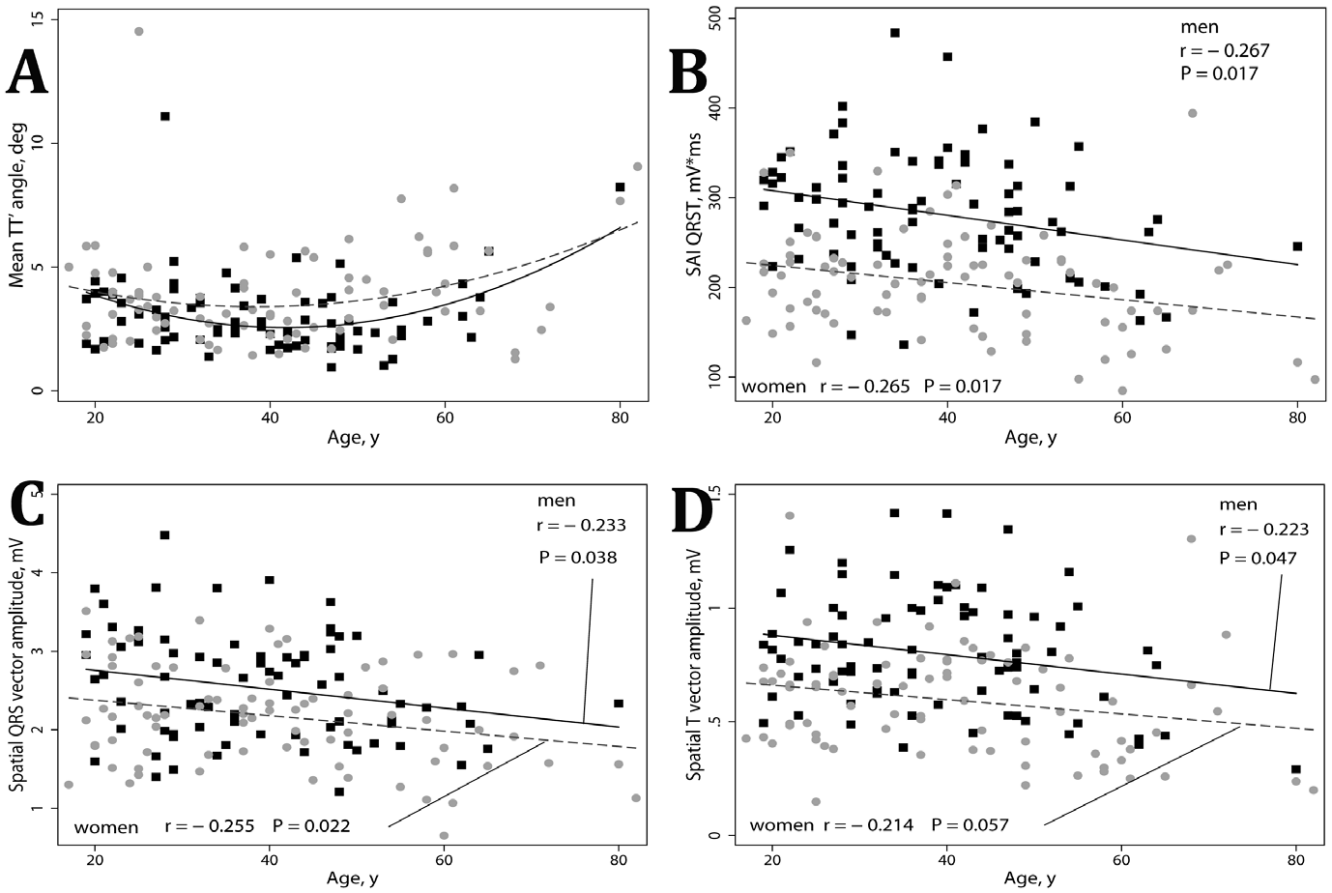
Correlation between ECG metrics and age. Scatterplots of (A) mean TT′ angle, (B) SAI QRST, (C) spatial QRS vector amplitude, (D) spatial T vector amplitude (Y) against age (X). Black squares represent male data points. Red circles represent female data points. A line of best fit is shown separately for men (black solid line) and women (red dashed line). Quadratic fit is used if non-linear relationship.

Age negatively correlated with amplitude VCG parameters and SAI QRST. Spatial QRS vector amplitude (r = −0.248; P = 0.002), spatial T vector amplitude (r = −0.221; P = 0.005) and SAI QRST (r = −0.253; P = 0.001) decreased with aging (Figure 6). No significant correlation of repolarization and depolarization lability with age was observed.

## Discussion

In this work, we provided detailed references (mean values, standard deviations and percentile ranges) of our 3D ECG temporal depolarization and repolarization variability parameters, interloop distance and SAI QRST in healthy adult men and women. We have found that SAI QRST and beat-to-beat variability in T-loop amplitude, area, and spatial TT′ angle, in addition to QTc and SVG, independently characterized electrophysiological differences between genders after adjustment for body size (BSA, BMI), systolic blood pressure, HR, heart rate variability and amplitude of spatial T vector. Importantly, we showed that after multivariate adjustment increased repolarization lability is associated with male, but not female sex. This fact was previously obscured by obvious gender differences in spatial QRS and T vector amplitudes. Spatial T and QRS vectors, T and QRS loops areas, SAI QRST, and SVG were significantly larger in men. Furthermore, repolarization lability negatively correlated with spatial T vector magnitude, which explained the seemingly larger repolarization lability in women observed in univariate analysis. Behavior of interloop distance VCG metric provided further insight in differences between genders. Repolarization lability did not correlate with age in healthy adults, whereas SAI QRST decreased with advanced age.

### Gender differences in repolarization lability

Increased repolarization lability is mechanistically linked with the risk of life-threatening ventricular arrhythmias [19]. However, quantitatively the degree of repolarization lability is small [19], and therefore the accuracy of its measurement is extremely important. Currently repolarization lability is almost uniformly characterized by variability of QT interval. However, QT interval is a measure of both depolarization and repolarization. Comparing key techniques of QT variability measurement (template-matching vs. based on QT interval measurement) showed that each technique has its limitations [20]. Moreover, we (Figure 5) and others [20] showed that repolarization lability inversely correlates with amplitude of spatial T vector (or T-wave), which might explain differences in reported predictive value of QT variability [21], [22] in clinical studies. Likely “the best” method of the measurement of repolarization lability depends on T-wave (or T-loop) size and morphology, and thus might vary in different populations. Repolarization could be characterized by several parameters beyond QT interval. On VCG repolarization is characterized by spatial T vector amplitude, T loop area, and T axis. In this study we expanded list of depolarization and repolarization variability parameters that could be measured by dynamic VCG and ECG. Several other approaches in studying variability of depolarization and repolarization were recently suggested [23], [24]. In this study we showed that various metrics of depolarization and repolarization variability are numerically very different (Figure 5). Importantly, a more efficient strategy might include a combination of techniques to quantify repolarization lability [4]. Mean TT′ angle stood out amongst all other metrics as the only parameter of repolarization lability which did not correlate with HR. We believe that beat-to-beat difference in T axis is the primary indicator of repolarization lability in humans, free from single lead bias. Absence of significant correlation between spatial TT′ angle and HR makes TT′ angle a particularly appealing metric for risk stratification, which will be tested in future studies.

VCGs have been shown to supply additional information to ECGs for repolarization characterization [25]. Combination of ECG and VCG analyses demonstrated a clear advantage [26]. However, VCG nowadays has limited use due to the belief that the techniques required are cumbersome and time consuming, and the results require complex interpretation. Fortunately, recent advancements in computer technologies have opened a new avenue for a VCG renaissance.

As previously noted, after multivariate adjustment healthy men, but not healthy women were characterized by larger repolarization lability. This observation seems contradictory to previous findings [27] only on the surface. Sexual dimorphism in ventricular repolarization was previously described [28] as steeper spatial ST-T vector voltage time trajectory in men as compared to women. Steep repolarization dynamics, observed by Lehmann and Yang [28] in men after stratification by heart rate and adjustment for age and a morphometric index of left ventricular mass might explain our findings.

Clinical studies showed that QT interval does not differ in boys and girls, but QT undergoes shortening in adolescent males [29] due to earlier onset of the fast phase 3 of left ventricular repolarization in males than in females [30]. Therefore, QT interval is not prolonged in healthy females - it is shortened in healthy males. Differences in presentation of ST segment and T wave on 12-lead ECG between adult men and women were described in detail by Surawicz et al [31] as “typical male” and “typical female” patterns. Lehmann and Yang [28] characterized sex differences in repolarization dynamics. It is clear that sex steroids likely mediate described differences in repolarization and its temporal variability. Androgens can affect repolarizing currents and modulate degree of heterogeneity of refractoriness as shown both in experiments [32]–[34] and clinical studies [35], [36].

### Gender differences in SVG and SAI QRST

Differences in amplitudes between genders are well known [28]. Normal VCG parameters have been described 50 years ago [37]. Our findings are consistent with previous studies of recorded [38] and derived VCG in apparently healthy Caucasians [39] and Chinese [40] subjects, which showed that the magnitude of the spatial QRS and T vectors decreased significantly with advancing age [38], [41], and was significantly larger in men in all age groups [42]. Observed range of SVG in our study was consistent with what was previously reported by Cortez et al [17]. Of note, we measured only spatial “peak” QRS-T angle, but not spatial “mean” QRS-T angle, which should be taken into account when interpreting our results [17]. Importantly, in agreement with results previously reported by Scherptong et al [43] based on data of a larger study of healthy individuals, we observed significantly wider spatial QRS-T angle in men regardless of selected measurement approach.

In addition to the previously discussed differences in sex steroids, differences in cardiac anatomy and in physiology between men and women explain the observed differences in ECG morphology [44]. It is apparent that body size strongly correlates with the size of the heart [45]. Interestingly, our study showed that even after adjustment for body size (BMI), systolic blood pressure, and QTc interval, both SAI QRST and SVG significantly associated with gender. The fact that SAI QRST and SVG strongly correlated with each other, but also both parameters significantly predicted sex in one multivariate regression model, means that these 2 parameters characterize somewhat different electrophysiological properties. The “ventricular gradient” concept was introduced by Wilson et al. in 1933 [46]. Subsequent studies [47], [48] showed that SVG reflects heterogeneity of the action potential morphologies in the ventricles of the heart [49]–[51]. SAI QRST is a relatively new and less studied ECG predictor of ventricular arrhythmia [6]–[8]. Future studies are needed to compare SAI QRST with SVG.

We observed a trend in a larger interloop distance in healthy men, in comparison to women (Table 2). We believe that large spatial T vector and wide interloop distance together with narrow spatial QRS-T angle might result in typical “early repolarization” ST-T presentation on ECG. As T vector amplitude decreases with age, “early repolarization” disappears with aging. Previously we showed that large interloop distance is associated with sustained ventricular arrhythmia in structural heart disease patients with implanted cardioverter-defibrillators [5]. In this study, interloop distance significantly correlated with heart rate and spatial T vector amplitude in women, but not in men (Figure 3D, 4D). Further studies of interloop distance might help further characterize gender differences in cardiac electrophysiology.

### Decreasing SAI QRST in healthy elderly: sign of cardiac atrophy?

Cardiac atrophy occurs in healthy elderly subjects [52] as part of the aging process. Mechanisms governing cardiac atrophy are poorly understood. Decreased physical activity with aging might cause ventricular unloading, which leads to cardiac atrophy in healthy adults. In our study, we observed a negative correlation between age and SAI QRST, which suggests that SAI QRST might be used to track the development of atrophy (or hypertrophy) over time. Future studies are needed to test this hypothesis.

### Limitations

This study sample size is relatively small. However, strict selection criteria resulted in the enrollment of a homogeneous group of healthy subjects. Despite the size, the study was sufficiently powered to detect gender differences. In this study we presented a spectrum of parameters, which characterize temporal variability of repolarization and depolarization. Future studies are needed to determine reproducibility, predictive value and practical utility of discussed metrics.

### Conclusions

ECG and VCG parameters of healthy men and women differ, which should be taken into account when developing gender-specific risk stratification. Healthy females are characterized by longer QTc, smaller SAI QRST, and lower repolarization lability, as compared to healthy males. SAI QRST decreases with advanced age, possibly reflecting atrophy in healthy elderly persons.

## Supporting Information

Movie S1 **Male VCG.** Movie shows QRS, T and P loops of consecutive 5 sinus beats of male study subject, rotated in different directions to demonstrate the 3D complexity of cardiac vector movement during the cardiac cycle.
(WMV)

Movie S2 **Female VCG.** Movie shows QRS, T and P loops of consecutive 5 sinus beats of female study subject, rotated in different directions to demonstrate the 3D complexity of cardiac vector movement during the cardiac cycle.
(WMV)
