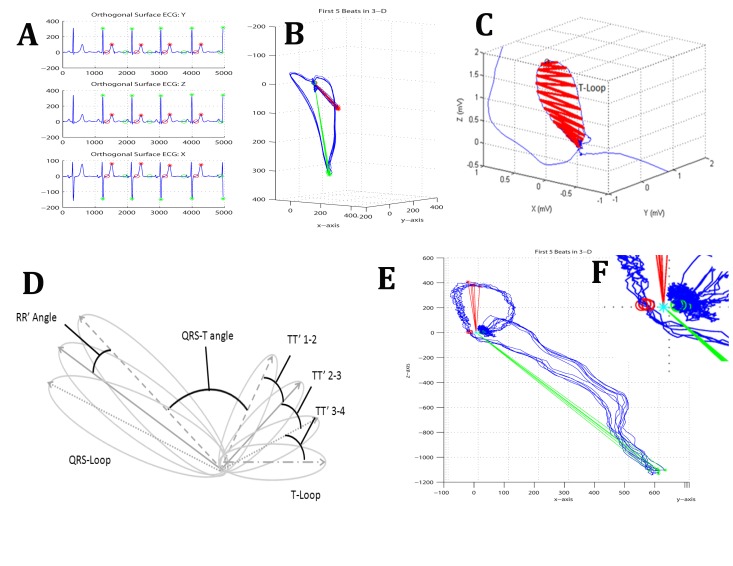# Correction: Comparison of Sum Absolute QRST Integral, and Temporal Variability in Depolarization and Repolarization, Measured by Dynamic Vectorcardiography Approach, in Healthy Men and Women

**DOI:** 10.1371/annotation/7ff61737-f005-41f6-b3bf-a80c104e1225

**Published:** 2013-10-30

**Authors:** Sanjoli Sur, Lichy Han, Larisa G. Tereshchenko

There is an error in the contents of Figure 1. Please view the correct Figure 1 here: 

**Figure:**